# Irradiation Damage Independent Deuterium Retention in WMoTaNbV

**DOI:** 10.3390/ma15207296

**Published:** 2022-10-19

**Authors:** Anna Liski, Tomi Vuoriheimo, Pasi Jalkanen, Kenichiro Mizohata, Eryang Lu, Jari Likonen, Jouni Heino, Kalle Heinola, Yevhen Zayachuk, Anna Widdowson, Ko-Kai Tseng, Che-Wei Tsai, Jien-Wei Yeh, Filip Tuomisto, Tommy Ahlgren

**Affiliations:** 1 Department of Physics, Helsinki Institute of Physics, University of Helsinki, P.O. Box 43, 00014 Helsinki, Finland; 2 VTT, Otakaari 3J, P.O. Box 1000, 02150 Espoo, Finland; 3 International Atomic Energy Agency, Vienna International Centre, P.O. Box 100, A-1400 Vienna, Austria; 4 Culham Science Centre, UK Atomic Energy Authority, Abingdon OX14 3DB, UK; 5 Department of Materials Science and Engineering, National Tsing Hua University, Hsinchu 30013, Taiwan; 6 High Entropy Materials Center, National Tsing Hua University, Hsinchu 30013, Taiwan

**Keywords:** high entropy alloy, metals, hydrogen, deuterium, fusion, elastic recoil, secondary ion, thermal desorption

## Abstract

High entropy alloys are a promising new class of metal alloys with outstanding radiation resistance and thermal stability. The interaction with hydrogen might, however, have desired (H storage) or undesired effects, such as hydrogen-induced embrittlement or tritium retention in the fusion reactor wall. High entropy alloy WMoTaNbV and bulk W samples were used to study the quantity of irradiation-induced trapping sites and properties of D retention by employing thermal desorption spectrometry, secondary ion mass spectrometry, and elastic recoil detection analysis. The D implantation was not found to create additional hydrogen traps in WMoTaNbV as it does in W, while 90 at% of implanted D is retained in WMoTaNbV, in contrast to 35 at% in W. Implantation created damage predicted by SRIM is 0.24 dpa in WMoTaNbV, calculated with a density of $6.044\times 10^{22}$ atoms/cm${}^{3}$. The depth of the maximum damage was 90 nm. An effective trapping energy for D in WMoTaNbV was found to be about 1.7 eV, and the D emission temperature was close to 700 °C.

## 1. Introduction

Thermonuclear fusion is a novel technology for clean and economical energy production associated with several environmental benefits. The technological challenges are under intensive research and development in order to have fusion power in full performance for energy production by the 2050s. One key challenge is related to the extreme conditions required for the fusion reactions, as hydrogen isotopes fuse in high temperatures under high magnetic fields and pressures and produce high fluxes of neutron and electromagnetic radiation. The conditions are demanding for the materials covering the plasma-facing wall of the reactor, and the ability of wall material to withstand this environment is a key issue for long-term energy production [1]. The current choices of materials for the first wall of the next-step fusion device ITER are beryllium and tungsten. Tungsten has good thermal conductivity, high sputtering resistance and low erosion yield, and retention of hydrogenic species in bulk W is low. However, energetic neutrons from the fusion reactions create hydrogen trapping damage in the armor materials, which may result in the accumulation of radioactive fusion fuel, tritium, in the wall components. Moreover, the tungsten plasma-facing surfaces can become brittle under irradiation and repeated thermal cycles during the reactor operation. Replacing tungsten with a material with similar properties in terms of sputtering and better irradiation resistance would be beneficial for decreasing the resulting wall damage and fusion fuel retention.

High entropy alloys (HEAs) are a new class of metal alloys characterized by multiple principal elements randomly distributed in the lattice [2]. Many such alloys exhibit high stability under elevated temperatures, and the variety of species present leads to multiple lattice-strengthening effects rendering the alloys auspicious new materials for extreme environment applications. The high entropy alloy chosen for this work is an equimolar mixture of refractory elements: WMoTaNbV with a high melting temperature of 2670 °C and fracture stress of 1370 MPa (1000 °C) [3,4].

The main requirements for a suitable reactor wall material are good thermal conductivity, high resistance to sputtering, erosion and neutron irradiation, and the ability to withstand variations in the temperature and magnetic field. The WMoNbTaV alloy can respond to the majority of these demands. The crystalline structure of the alloy is affected by multiple strengthening effects and secondary phases, generating exceptional hardness and mechanical durability [5]. Ideally, one feature of an appropriate wall material would also be the lack of hydrogen trapping defects created under irradiation, leading to low retention of fusion fuel. In this study, we quantify the amount of retained hydrogen and deuterium in WMoTaNbV and compare results with those of W, the current material of choice. Deuterium is used to model tritium retention properties in order to avoid radiological hazards. Despite the differences in the masses of these hydrogen isotopes, their chemical properties are similar, and their trapping behavior in metals is comparable. D is introduced to the material by relatively high-energy implantation (20 keV/D) that leads to irradiation damage in the HEA and W samples. This is expected to lead to high D retention due to the defects created during implantation, as is the case in W. Interestingly, in WMoTaNbV, the D retention appears independent of the irradiation-induced damage.

## 2. Experimental

### 2.1. Sample Preparation

The WMoTaNbV samples were prepared at the High Entropy Materials Center of National Tsing Hua University by vacuum arc melting [6]. The details of the synthesis procedure are given elsewhere [7]. The concentrations of components in the alloy are reported to be Mo (20.7 at%), Nb (20.3 at%), Ta (20.4 at%), V (19.2 at%) and W (19.4 at%). The WMoTaNbV samples were received cut with dimensions 5 × 5 × 1 mm${}^{3}$. The bulk W samples were cut from a polycrystalline W sheet (Goodfellow, 99.95 wt% ). The dimensions of the pieces are 10 × 10 × 1 mm${}^{3}$.

The surfaces of the WMoTaNbV and W samples were mirror polished. Rough polishing was performed using Silicon Carbide grinding paper (Buehler, Uzwil, Switzerland) finished with monocrystalline diamond suspension (Akasel, Aka-mono, Roskilde, Denmark) of particle sizes down to 50 nm. The surface roughness was measured with a stylus profilometer (KLA-Tencor P-15) and was Ra ≈ 0.3 μm. Hydrogen and other gaseous impurities were released from the materials by pre-annealing in a quartz tube furnace at 1000 °C (2 h) in a vacuum below $10^{-4}$ Pa.

### 2.2. Positron Lifetime Spectroscopy

Positron lifetime spectroscopy was employed to quantify the presence of vacancy-type defects in W and WMoTaNbV, as they are known to be efficient hydrogen traps in W. A digital positron lifetime spectrometer with 250 ps Gaussian timing resolution (full width at half maximum) was used to collect $10^{6}$ annihilation events in each spectrum. The data were analyzed by fitting a sum of exponential decay components to the spectra. For more details of the method, see Ref. [8]. A 10-MeV proton irradiation was used for the comparison of damage production in both materials.

### 2.3. D Implantation

A 500 kV accelerator was used at the University of Helsinki for the D implantations. Samples were fixed on an aluminum holder perpendicularly to the D${}_{2}^{+}$ beam, which was swept over the whole sample surface area to reach a uniform D${}_{2}$ beam exposure over the surface. The implantation was performed at room temperature with low D${}_{2}$ flux in order to avoid any excess heating. The flux density was approximately 8 $\times \;10^{12}$ D/(cm${}^{2}$s), taking less than 2 h to reach the desired dose. The ion energy used for the implantations was 20 keV/D and the fluence 5 $\times \;10^{16}$ D/cm${}^{2}$. The backscattered D from the samples during implantation was calculated using SRIM simulations [9] as 8.4% and 15.1% for WMoTaNbV and bulk W, respectively. Thus, the comparable bulk fluences were assessed to be 4.58 $\times \;10^{16}$ D/cm${}^{2}$ in WMoTaNbV and 4.25 $\times \;10^{16}$ D/cm${}^{2}$ in W. The damage created by implantation was estimated from SRIM, and it was 0.24 dpa in WMoTaNbV and 0.20 dpa in W. The densities were $6.044\times 10^{22}$ atoms/cm${}^{3}$ and $6.338\times 10^{22}$ atoms/cm${}^{3}$ for WMoTaNbV and W, respectively.

### 2.4. Elastic Recoil Detection Analysis (ERDA)

ERDA measurements were performed using the 5 MV tandem accelerator located at the University of Helsinki. Foil-ERDA were used for measuring the retained D concentrations in both WMoTaNbV and bulk W. The incident ion was silicon (${}^{28}$Si${}^{5+}$) with an energy 24 MeV per ion. Si was chosen to maximize the recoil yield and to prevent any overlap of the close to surface D and H recoil signals. The incident Si ion angle was $\alpha_{\mathrm{in}}$ = 60°, and the recoil products were measured from an angle of $\alpha_{\mathrm{out}}$ = 75° from the surface normal. The detector was positioned at 5 cm distance from the target, and its detection angle was limited with a 2 × 7 mm${}^{2}$ collimator. Havar stopping foil with 4.0 μm thickness was used to filter out the scattered Si and heavier recoils. The nominal resolution of the detector was 15 keV.

### 2.5. Secondary Ion Mass Spectrometry (SIMS)

The hydrogen profiles were studied with SIMS using a double focusing magnetic sector instrument VG Ionex IX-70S at VTT Technical Research Centre of Finland. A 12 keV Cs${}^{+}$ primary beam with 100 nA current was raster-scanned over an area of 300 × 300 μm${}^{2}$. A 10% electronic gate was used to avoid ejecting the particles from the edges of the crater created by SIMS. The intensities of the negative secondary ions at mass-to-charge ratios (m/q) of 1 (H${}^{+}$), 2 (D${}^{+}$), 12 (C${}^{+}$), 51 (V${}^{+}$), 93 (Nb${}^{+}$), 98 (Mo${}^{+}$), 181 (Ta${}^{+}$) and 184 (W${}^{+}$) were measured as a function of time. After each measurement, the depth of the crater was measured with a profilometer. The D amounts were quantified by normalizing the SIMS results to the total D concentrations determined with ERDA.

### 2.6. Thermal Desorption Measurements

H/D release from the investigated samples was measured by means of thermal desorption spectrometry (TDS). The TDS measurements were performed at Culham Centre for Fusion Energy using Hiden Analytical Ltd TPD Workstation Type 640100 (Warringtom, UK). A detailed description of the facility can be found in Ref. [10].

In the experiments, samples were loaded onto a molybdenum heater stage inside a vacuum chamber with a baseline pressure of ∼2.7 $\times \;10^{-7}$ Pa. Samples were heated from their back side, and a line-of-sight quadrupole mass spectrometer (QMS) detected the signals of selected molecular species desorbing from the ion-implanted sample and recorded the corresponding m/q ratio as a function of time. The TDS background measurement was performed prior to the annealing experiment and was subtracted from the experimental results of corresponding mass signals. m/q signals of 3 and 4, corresponding to HD${}^{+}$ and D${}_{2}^{+}$ molecules, respectively, were monitored and used for quantifying the deuterium release. These signals were calibrated using H${}_{2}$ and D${}_{2}$-calibrated leaks, where the calibration factor for the HD signal was obtained as an average of the H${}_{2}$ and D${}_{2}$ calibration factors. The total D atomic release is calculated as a sum of HD and 2·D${}_{2}$. Signal m/q=2 (H${}_{2}^{+}$ molecules) was quantified using an H${}_{2}$-calibrated leak. Signals m/q=18 (H${}_{2}$O), 19 (HDO), 20 (D${}_{2}$O), 28 (N${}_{2}$), 32 (O${}_{2}$) and 44 (CO${}_{2}$) were unquantified but used in monitoring for the presence of any contaminants.

During the measurement, the sample was heated from room temperature to 1000 °C at a constant ramp rate and then held at the maximum temperature for 1 h. Ramp rates for annealing were varied and values of 5, 10 and 20 °C/min were used.

## 3. Results

### 3.1. D Concentration Profiles

**Figure 1:**
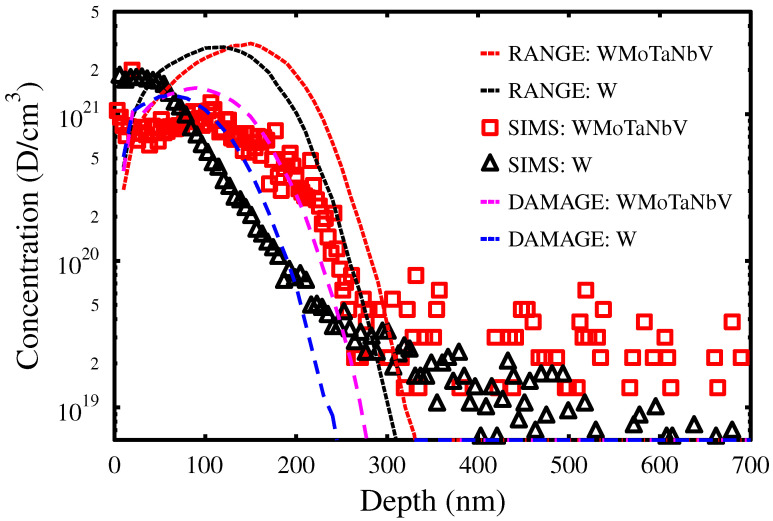
Concentration profiles of D in WMoTaNbV and W. The profiles were measured by SIMS and calibrated with the total D amounts from ERDA. The SRIM profiles show approximately how the profiles would look if there were no D diffusion. Displacement threshold energies used were W = 90 eV, Mo = 65 eV, Nb = 78 eV, Ta = 90 eV and V = 57 eV [11]. The units of SRIM-calculated damage profiles are arbitrary.

The total amount of retained D in the implantation region was obtained by combining the results measured with SIMS and ERDA techniques. SIMS D distributions were calibrated using the total D amounts measured by ERDA up to depths of ∼700 nm. The resulting D profiles are shown in Figure 1. The concentration maximum of D in W is located between the sample surface and the depth of 70 nm, while in WMoTaNbV, the highest D concentrations are found at the surface and at about 100 nm. The D profile in WMoTaNbV extends deeper than in W partly due to the lighter elements present in the HEA alloy.

Both materials show significantly less D in the implantation region than estimated from the SRIM profiles. Hence, diffusion of D already occurs at room temperature in both materials.

The large reduction in the D at the implanted depths also indicates that the number of D trapping centers is initially low and that these are not excessively produced during implantation. In bulk W, D is known to be efficiently trapped in monovacancies and small vacancy clusters produced during the ion implantation [12]. This is also evident from Figure 1, where the D in W is seen to be trapped in a region close to the surface, where most of the implantation-induced mono-vacancies are produced. However, in WMoTaNbV, D does not seem to be trapped mainly in the irradiation-induced defects since the measured D concentration close to the surface, up to about 200 nm, is quite constant, around 8 $\times \;10^{20}$ D/cm${}^{3}$, and significantly lower than in W. A backscattering corrected implanted dose of D was compared with experimentally measured quantities resulting in 33.6% and 38.5% D retention near the surface (0–700 nm) in WMoTaNbV and W, respectively. Details are given in Table 1.

### 3.2. Vacancy Defects and Irradiation Damage

Positron lifetime was measured at room temperature in the W and WMoTaNbV before and after irradiation with 10-MeV protons (fluence 1 $\times \;10^{16}$ cm${}^{-2}$), which is known to produce mono-vacancies [13]. Before irradiation, the positron lifetime spectra exhibit only one decay component in both materials. The values are 118 ps for W and 126 ps for WMoTaNbV, which correspond to the lifetime in the lattice, indicating that the concentrations of vacancy-type defects are at or below the detection limit on the method, about $10^{15}$ cm${}^{-3}$ [8,14,15]. After irradiation, the average lifetime increases to 142 ps in W and to 167 ps in WMoTaNbV, and in both materials, a second lifetime component of 180 ps is detected. This lifetime component corresponds to mono-vacancies in W [13]. The more significant increase in the average lifetime in WMoTaNbV indicates that a larger fraction of the mono-vacancies survives the recombination processes during irradiation than in W. Hence, vacancy-type irradiation damage in WMoTaNbV is at least as important as in W.

### 3.3. Trapping Dynamics

**Figure 2:**
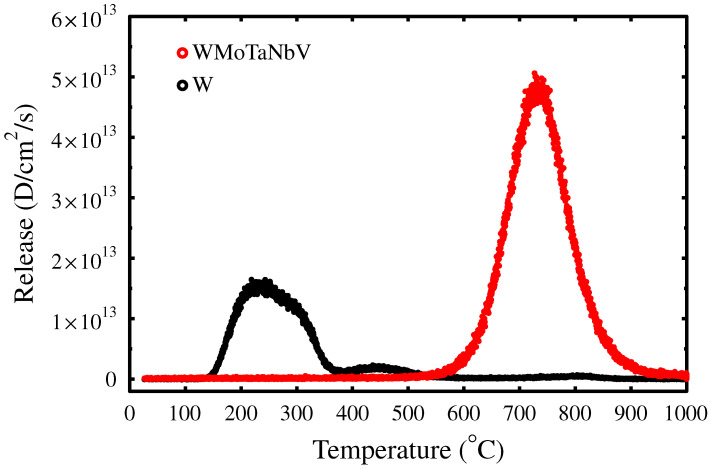
Thermal release D signals from WMoTaNbV and W with 10 °C/min heating rate.

The trapping behavior of D in WMoTaNbV and W was determined by TDS. The amounts of released D were calculated using signals D${}_{2}$ and HD. Three samples of WMoTaNbV were heated from room temperature up to 1000 °C with heating rates of 5, 10, and 20 °C/min. The W sample was heated up to the same temperature with a rate of 10 °C/min. D was observed to be released from W in several temperature stages, while the release from WMoTaNbV occurred in a single wide emission peak. Calibrated desorption signals with a ramp of 10 °C/min are shown in Figure 2, and the retention results are summarized in Table 1.

**Figure 3:**
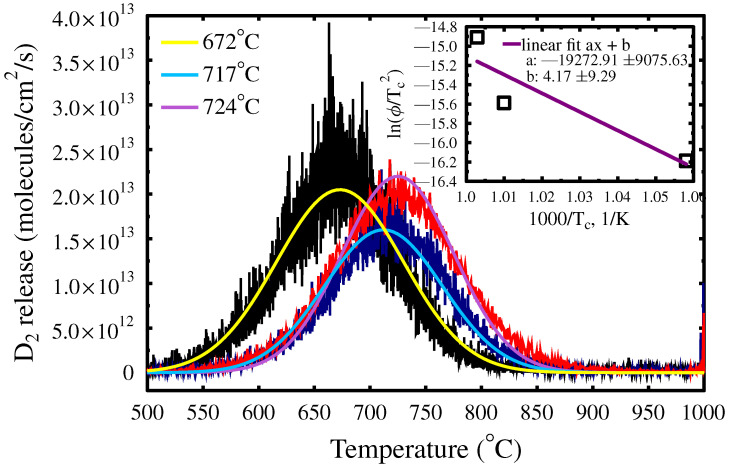
The three thermal desorption measurements from the WMoTaNbV alloy with heating rates of 5, 10, and 20 °C per minute. The D release peak positions correspond to the trapping energy of 1.66 ± 0.78 eV.

The TDS results show that D is released from WMoTaNbV at high temperatures around approximately 700 °C and is freed in a single emission peak, indicating the trapping to a defect of a specific type or trapping to numerous different traps with similar trapping energies. The trapping energy is determined from equation

(1) $$\frac{E_{trap}}{kT_{c}^{2}}=\frac{\upsilon }{\phi e^{-E_{trap}/kT_{c}}},$$

where υ is the particle’s frequency, *k* is the Boltzmann constant, ϕ the linear heating rate of the sample, and $T_{c}$ corresponds to temperature with the maximum release rate [16,17]. The release temperatures were determined by fitting a normal distribution over the emission peaks with the maximum coinciding with temperatures 672, 717 and 724 °C, ordered from smallest to the largest heating ramp. The trapping energy $E_{trap}$ was obtained from Equation (1) with a linear fit. The fit is presented in the inset of Figure 3, where the slope is $E_{trap}/k$, giving a trapping energy of 1.7 ± 0.8 eV. The constant *b* comes from the remaining part of the equation −ln(E/kυ) and is, therefore, not dependent on the temperature nor the heating rate.

Desorption measurements show that D retention in W (Table 1) is about 35%, while in WMoTaNbV, the D retention is over 90%, and the majority of the implanted D is retained within the bulk of WMoTaNbV alloy (Table 1). This result differs from kinetic rate equation (kRE) simulations [12,18], which predicted the retention to be between 35% and 46% depending on the concentration of D traps.

High D retention observed in WMoTaNbV could have several explanations: there might be an impurity barrier at the surface that prevents D from reaching the surface. There could be an energy barrier from the bulk to the surface, or the D solution into WMoTaNbV might be exothermic (negative solution energy). This fundamental issue is further explored in the next section.

### 3.4. H Trapping to Irradiation-Induced Defects

Figure 3 shows that all D is released from WMoTaNbV in a single wide emission peak. This means that the close to surface D (Figure 1), which accounts for about 34% of the total D amount in the sample (Table 1), is detrapped at the same temperature as the rest of the D deeper in the sample. This indicates that implantation damage in WMoTaNbV does not trap D at room temperature.

**Figure 4:**
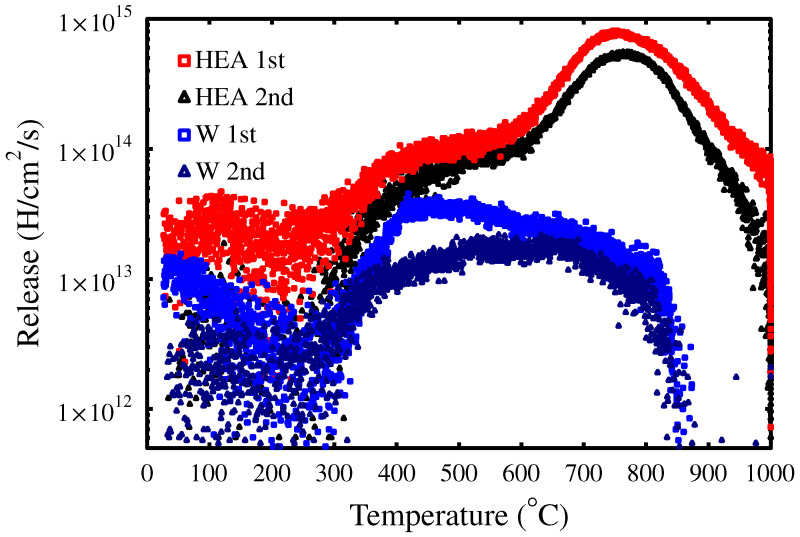
Hydrogen release signals from WMoTaNbV and W during the TDS measurements. The second measurement was taken after the samples had spent several weeks in the NTP conditions. Both materials reabsorb hydrogen from the atmosphere, WMoTaNbV significantly more than W.

To further explore the trapping dynamics, the TDS measurements were performed twice on the same samples of W and WMoTaNbV by examining only the hydrogen (H) concentrations. The hydrogen releases are presented in Figure 4. The samples were implanted with D, and during the first TDS measurement, all the hydrogen and its isotopes were removed. In total, the WMoTaNbV alloy released 4.34 × 10${}^{17}$ at/cm${}^{2}$ and W 2.49 × 10${}^{16}$ at/cm${}^{2}$ of hydrogen in the first run. The samples were then left in the NTP conditions for the duration of several weeks and then re-measured with the same heating rate as before, 10 °C/min for both WMoTaNbV and W samples. During the second run, the hydrogen releases were roughly half of the first run: 2.37 × 10${}^{17}$ at/cm${}^{2}$ and 8.84 × 10${}^{15}$ at/cm${}^{2}$ for WMoTaNbV and W, respectively. A large proportion of hydrogen has been reintroduced into WMoTaNbV, and a smaller proportion into W as well. No diffusion barrier from the surface to the bulk seems to be present in WMoTaNbV. Further, the release peak of WMoTaNbV (Figure 4) has a similar shape in both of the TDS measurements, suggesting that the traps survive the heating and that the absorbed hydrogen continues to be trapped in the same sites as in the first measurement.

In comparison, W shows partly different shapes of the emission peaks between the runs. The low temperature (between 350–600 °C) H desorption peaks have decreased, indicating that the D irradiation-induced monovacancies and small vacancy clusters, with smaller H binding energy, have decreased during the first TDS run- The migration barrier for monovacancies in W is about 1.8 eV, and the monovacancies start to diffuse at about 300 °C [13,19,20,21]. The H desorption has a very similar shape between 600 and 900 °C, which could be H-trapped to large vacancy clusters, grain boundaries and impurities.

## 4. Discussion

The H/D trapping in WMoTaNbV is unlikely associated with small vacancies as those are created at high concentrations during irradiation in the vicinity of the surface, and they do not pre-exist in the samples. Additionally, the literature values for hydrogen trapping energy to monovacancies in the Ta, Nb, Mo and V are significantly lower, as shown in Table 2 based on data from [22,23]. The trapping energy to the monovacancies in W has a similar magnitude (1.6 eV) to the D trapping in WMoTaNbV (1.7 eV). However, only a small fraction of all mono-vacancies have clear W mono-vacancy character in a five-element random alloy.

The D release from WMoTaNbV occurs as a one-emission peak but with a relatively wide trapping energy distribution (1.7±0.8 eV). This indicates a large variation in the D trapping sites, suggesting that D is trapped in features closely related to the random alloy matrix. It is also possible that D is acting as an alloying element or trapped at impurity atoms embedded in the bulk. Due to manufacturing process of melting, small amounts of common impurity atoms such as C, N, and O could be found in the final alloy as well. For example, the substitutional carbon has a relatively strong trapping energy of about 1.5 eV in W [25]. This suggests that in specific environments, C impurities can act as strong traps. It is worth noting that, in general, high entropy alloys accommodate small-atom interstitials much more efficiently than dilute metal alloys thanks to the wide lattice distortion distributions [26,27]. Nevertheless, it is evident that mono-vacancies are not efficient H/D traps in WMoTaNbV.

The traps remain after the TDS heating to 1000 °C in WMoTaNbV. In W, the traps remain partially after the heating as well, but part of the implantation-induced monovacancies and other small vacancy clusters are removed, changing the TDS release profile. Both materials show the trapping of hydrogen from the atmosphere at room temperature, but for WMoTaNbV, the amounts absorbed are much higher. This suggests that the solution energy of WMoTaNbV could be negative, but the magnitude of the trapping energy, ∼1.7 eV, would still suggest deep traps present in the material. This high trapping energy is also in line with observations of disorder-induced unique elemental diffusion properties in high entropy alloys [28,29]. Further studies are needed to determine the nature of the traps or the magnitude of solution energy in WMoTaNbV.

## 5. Conclusions

The room temperature hydrogen diffusion is rapid in both materials—WMoTaNbV and W. The majority of the implanted D is retained in the bulk of the WMoTaNbV sample, and the trapping occurs with a high energy of about 1.7±0.8 eV. The trapping mechanism is unlikely to be the same as in W since H/D is not binding in the small irradiation-induced, vacancies near the surface. Instead, it could be that D is forming a chemical bond, trapped to impurities or trapped at major structural defects such as grain boundaries. It is also possible that the wide lattice distortion distribution in the high entropy alloy contains natural trapping sites for hydrogen even without the presence of defects. Further investigation is required to reveal the detailed trapping mechanisms of H/D in WMoTaNbV.

From the perspective of applicability as a fusion first-wall material, the high hydrogen retention is non-ideal. However, irradiation does not seem to produce defects in WMoTaNbV that trap hydrogen, contrary to all other considered first-wall materials. Therefore, if WMoTaNbV proves itself to be a superior material with regard to all the other features, it is possible to minimize the retention problem by using only thin layers of coverage in the plasma-facing wall.

## Figures and Tables

**Table 1:** Quantities of D measured from WMoTaNbV and W with elastic recoil detection and thermal desorption. * Corrected for backscattering during the implantation, a correction factor was obtained from SRIM. ** Compared with backscattering corrected implanted dose.

		ERDA		TDS		
	Impl. Dose *	**Abs. Reten.**	Rel. Reten. **	**Heating Rate**	**Abs. Reten.**	Rel. Reten. **
W	4.25 $\times \;10^{16}$	1.64 $\times \;10^{16}$	38.5%	10 °C/min	1.50 $\times \;10^{16}$	35.3%
HEA				20 °C/min	4.14 $\times \;10^{16}$	90.4%
HEA	4.58 $\times \;10^{16}$	1.54 $\times \;10^{16}$	33.6%	10 °C/min	4.20 $\times \;10^{16}$	91.7%
HEA				5 °C/min	4.33 $\times \;10^{16}$	94.5%

**Table 2:** Hydrogen trapping energies to monovacancy by element. Energies are calculated by first principles, including ZPE correction.

	W (eV) [22]	Mo (eV) [24]	Ta (eV) [23]	Nb (eV) [23]	V (eV) [23]
1H	1.60	1.25	0.38	0.41	0.44
2H	1.57	1.25	0.43	0.47	0.53
3H	1.39	0.97	0.33	0.30	0.43
4H	1.28	0.89	0.26	0.25	0.34
5H	1.17	0.75	0.35	0.24	0.33
6H	0.64	0.54	0.13	0.09	0.28

## Data Availability

The authors declare that the data supporting this study are available from the corresponding author upon request.

## Abbreviations

The following abbreviations are used in this manuscript:

HEA	high entropy alloy
ERDA	elastic recoil detection analysis
SIMS	secondary ion mass spectrometry
TDS	thermal desorption spectrometry
